# Fibroblast Growth Factor-9 Enhances M2 Macrophage Differentiation and Attenuates Adverse Cardiac Remodeling in the Infarcted Diabetic Heart

**DOI:** 10.1371/journal.pone.0120739

**Published:** 2015-03-13

**Authors:** Dinender K. Singla, Reetu D. Singla, Latifa S. Abdelli, Carley Glass

**Affiliations:** Biomolecular Science Center, Burnett School of Biomedical Sciences, College of Medicine, University of Central Florida, Orlando, Florida, United States of America; Georgia Regents University, UNITED STATES

## Abstract

Inflammation has been implicated as a perpetrator of diabetes and its associated complications. Monocytes, key mediators of inflammation, differentiate into pro-inflammatory M1 macrophages and anti-inflammatory M2 macrophages upon infiltration of damaged tissue. However, the inflammatory cell types, which propagate diabetes progression and consequential adverse disorders, remain unclear. The current study was undertaken to assess monocyte infiltration and the role of fibroblast growth factor-9 (FGF-9) on monocyte to macrophage differentiation and cardioprotection in the diabetic infarcted heart. Db/db diabetic mice were assigned to sham, myocardial infarction (MI), and MI+FGF-9 groups. MI was induced by permanent coronary artery ligation and animals were subjected to 2D transthoracic echocardiography two weeks post-surgery. Immunohistochemical and immunoassay results from heart samples collected suggest significantly increased infiltration of monocytes (Mean ± SEM; MI: 2.02% ± 0.23% vs. Sham 0.75% ± 0.07%; p<0.05) and associated pro-inflammatory cytokines (TNF-α, MCP-1, and IL-6), adverse cardiac remodeling (Mean ± SEM; MI: 33% ± 3.04% vs. Sham 2.2% ± 0.33%; p<0.05), and left ventricular dysfunction (Mean ± SEM; MI: 35.4% ± 1.25% vs. Sham 49.19% ± 1.07%; p<0.05) in the MI group. Importantly, treatment of diabetic infarcted myocardium with FGF-9 resulted in significantly decreased monocyte infiltration (Mean ± SEM; MI+FGF-9: 1.39% ± 0.1% vs. MI: 2.02% ± 0.23%; p<0.05), increased M2 macrophage differentiation (Mean ± SEM; MI+FGF-9: 4.82% ± 0.86% vs. MI: 0.85% ± 0.3%; p<0.05) and associated anti-inflammatory cytokines (IL-10 and IL-1RA), reduced adverse remodeling (Mean ± SEM; MI+FGF-9: 11.59% ± 1.2% vs. MI: 33% ± 3.04%; p<0.05), and improved cardiac function (Fractional shortening, Mean ± SEM; MI+FGF-9: 41.51% ± 1.68% vs. MI: 35.4% ± 1.25%; p<0.05). In conclusion, our data suggest FGF-9 possesses novel therapeutic potential in its ability to mediate monocyte to M2 differentiation and confer cardiac protection in the post-MI diabetic heart.

## Introduction

Diabetes mellitus is a metabolic disorder, which is characterized by hyperglycemia consequent to the body’s inability to either produce any or sufficient quantities of insulin. Diabetic patients, with chronic uncontrolled glycemic indexes, are at a higher risk for developing secondary disorders including cardiovascular disease, retinopathy, nephropathy, and neuropathy [1–4]. Data suggest inflammation plays a critical role in the development and progression of diabetes and associated complications through infiltration of immune cells and upregulated monocytic activity [5–8].

Monocytes are key mediators of inflammation and upon infiltration into damaged tissue, undergo reprogramming yielding two distinct macrophage phenotypes: pro-inflammatory M1 macrophages, which promote cytotoxic effects through secretion of inflammatory cytokines including TNF-α, MCP-1, and IL-6 and anti-inflammatory M2 macrophages, which are implicated in the mediation and resolution of inflammation, in part, through secretion of anti-inflammatory cytokines including IL-10 and IL-1RA [9]. The ratio of M1:M2 macrophages has been identified as a key determinant of the severity and progression of inflammatory propagated pathologies [10, 11]. In fact, several investigations using obese, insulin resistant db/db diabetic mice, have shown dysregulated monocyte/macrophage infiltration and retention with subsequent enhanced pro-inflammatory cytokine release in the aorta and pancreatic islets and during wound healing [5–8]. However, whether monocytic infiltration and M1/M2 macrophage differentiation outcomes play a role in adverse cardiac remodeling following MI in the diabetic heart has yet to be evaluated and remains largely unknown.

FGFs comprise a family of twenty-two polypeptide growth factors that span a myriad of biological functions including those involved in embryonic development, tissue morphogenesis, and post-natal physiological homeostasis [12, 13]. Specifically, FGF-9, initially isolated and identified in culture media obtained from glial cells, has been shown to play an important role, like other FGF family members, in variegated cellular processes, spanning multiple cell types and tissues [14]. Such functions include neuronal cell growth and development, inner ear morphogenesis, wound induced hair follicle neogenesis, joint development, neovasculogenesis, and testicular embryogenesis [15–20]. Recently, data has been presented suggesting conditional expression of FGF-9 in the post-MI heart promoted vascularization and left ventricular hypertrophy, improved systolic function, and reduced subsequent death [21]. However, whether FGF-9 promotes cardioprotection in the post-MI diabetic heart through promotion of anti-inflammatory mechanisms (enhanced M2 macrophage differentiation and anti-inflammatory cytokine release) and diminished pro-inflammatory responses (reduced M1 macrophage differentiation and pro-inflammatory cytokine secretion) remained elusive. The current study was undertaken to evaluate the potential role of FGF-9 in directing monocyte to M2 macrophage differentiation and its associated inhibition of adverse cardiac remodeling in the post-MI diabetic heart.

## Materials and Methods

### Coronary Artery Ligation and FGF-9 Treatment

All animal protocols were reviewed and approved by the University of Central Florida Institutional Animal Care and Use Committee (IACUC). Male and female db/db diabetic mice, 8–10 weeks old were divided into 3 groups (n = 7–9 animals/group): Sham, MI, and MI+FGF-9 (1ng/10μl 0.1% BSA in 1X PBS). As previously detailed, mice were anesthetized using endo-tracheal delivered isoflurane and subjected to a left thoracotomy [22, 23]. The left anterior descending coronary artery was permanently ligated using a 7.0 polypropylene suture in the MI and MI+FGF-9 groups. The sham-operated group was subjected to all procedures excluding permanent coronary artery ligation. Following ligation, the MI+FGF-9 group was injected with FGF-9 into two independent sites (1 ng FGF-9/injection prepared as aforementioned for a total dose of 2 ng FGF-9) in the peri-infarct region using a 29-guage floating needle. Two weeks post-surgery, animals were sacrificed using 4% inhalatory isoflurane for 10 mins followed by cervical dislocation. Hearts from all groups were removed, washed in phosphate buffered saline (PBS), and preserved in either RNA later or formalin solution for future evaluations.

### Tissue Section Preparation and Histopathology

Formalin preserved hearts were embedded in paraffin, cut into 5 μm serial sections, and placed on Colorfrost plus-plus slides as previously published [24]. Following deparaffinization and rehydration, sections were stained with Masson’s Trichrome to visualize fibrosis (blue area). Infarct size (%) was quantified by measuring (infarct area (mm^2^)/total area (mm^2^))*100. Interstitial fibrosis (IF) was quantified by evaluating the total blue area per mm^2^ with NIH Image J software. Infarct size (%) and interstitial fibrosis were measured in 1–2 sections (4 images captured per section and calculated data averaged per section) from different animals. The number of animals/group are listed in the figure legends.

### Immunohistochemistry—Monocyte and Macrophage Identification

Sections were subjected to heat-induced epitope retrieval (HIER) for 20 mins followed by blocking with 10% normal goat serum (Vector Labs) for one hr. Slides were incubated with primary anti-rabbit antibodies either against CD14 (monocytes, Abbiotec, #25156, 1:50), iNOS (M1 macrophages, Abcam, #ab129372, 1:50), or CD206 (M2 macrophages, Abcam, #ab64693, 1:50) overnight at 4°C. Slides were then washed and incubated with appropriate secondary antibodies for one hr at room temperature. All slides were additionally double stained with primary (and appropriate secondary) antibodies against sarcomeric α-actin (Src α-actin) (cardiac myocyte, Sigma, #A2172, 1:30). Src α-actin staining was carried out using M.O.M. Immunodetection kit reagents following the Multiple Immunofluorescent Labeling protocol provided (Vector Laboratories, #FMK-2201). Sections were counter stained with mounting medium containing DAPI (4’, 6-diamidino-2-phenylindole) (Vector Labs) and cover-slipped. Four images per section (1–2 sections, n = 5 animals/group) were taken for data quantification using an Olympic IX-70 fluorescent microscope and representative images were captured using a confocal microscope. Cells positive for CD14, iNOS, or CD206 were independently counted, divided by the total number of nuclei, and expressed as a percent of positive cells/section.

### Real-time RT-PCR

Total RNA was prepared from heart homogenates according to the manufacturer’s instructions using TRI Reagent RNA Isolation Reagent (Sigma-Aldrich). RNA was reversely transcribed using iScript Select cDNA Synthesis Kit (Bio-Rad) with Random primers, and real-time PCR was carried out using a C1000 Thermal Cycler (Bio-Rad). Real-time PCR analysis was performed using SYBR Green (Bio-Rad) and normalized to GAPDH. Relative expression was assessed by the comparative CT method correcting for amplification efficiency of the primers. Primers used for real-time PCR were as follows:

- TNFα: sense primer, 5′-CACAGAAAGCATGATCCGCGACGT-3′;
- Antisense primer, 5′-CGGCAGAGAGGAGGTTGACTTTCT-3′;
- IL-6: sense primer, 5′-TCCAGTTGCCTTCTTGGGAC-3′;
- Antisense primer, 5′-GTACTCCAGAAGACCAGAGG-3′;
- IL-10: sense primer, 5′-TGGCCCAGAAATCAAGGAGC-3′;
- Antisense primer, 5′-CAGCAGACTCAATACACACT-3′;
- GAPDH: sense primer, 5′-AACGACCCCTTCATTGAC-3′;
- Antisense primer, 5′-TCCACGACATACTCAGCAC-3′;
- Arg1: sense primer, 5′-CTCCAAGCCAAAGTCCTTAGAG-3′;
- Antisense primer, 5′-AGGAGCTGTCATTAGGGACATC-3′;

Data is presented as relative fold increase against the sham group.

### Pro- and Anti-Inflammatory Cytokine Analysis by ELISA

Secreted pro-inflammatory cytokines (TNF-α#ELM-TNFalpha-001, #MCP-1 #ELM-MCP-001, and IL-6 #ELM-IL6-001) and anti-inflammatory cytokines (IL-10 #ELM-IL10-001 and IL-1RA #ELM-IL1RA-001) were quantified using commercially available RayBiotech ELISA kits. In brief, heart tissue was homogenized in RIPA buffer containing PMSF, sodium orthovandate, and sodium fluoride. Following centrifugation, supernatant was collected and protein concentration was quantified using the Bradford assay. All ELISAs were performed following manufacturer’s instructions and the resulting colorimetric reactions were measured at 450 nm. Results were normalized to protein concentration and data is presented as pg/ml.

### Cardiac Function Analysis

Two weeks post-sham or MI surgery, transthoracic 2D echocardiography was performed using the 5500 Ultrasound System as previously described [25]. A 15-6L hockey stick transducer was used to capture M-mode images of the left ventricle for left ventricular internal dimension-diastole (LVIDd), left ventricular internal dimension-systole (LVIDs), fractional shortening (FS, ((LVIDd-LVIDs)/LVIDd x 100)), left ventricular volume at end diastole (EDV), left ventricular volume at end systole (ESV), and ejection fraction (EF, ((EDV-ESV)/EDV x 100)) calculations.

### Statistical Analysis

Statistical analysis of data was performed using one-way analysis of variance (ANOVA) followed by the Tukey Test. Data is presented as a mean ± SEM with p<0.05 considered statistically significant.

## Results

### Infarct Size and Fibrosis Blunted Following FGF-9 Treatment in Post-MI Diabetic Myocardium

**Fig 1:**
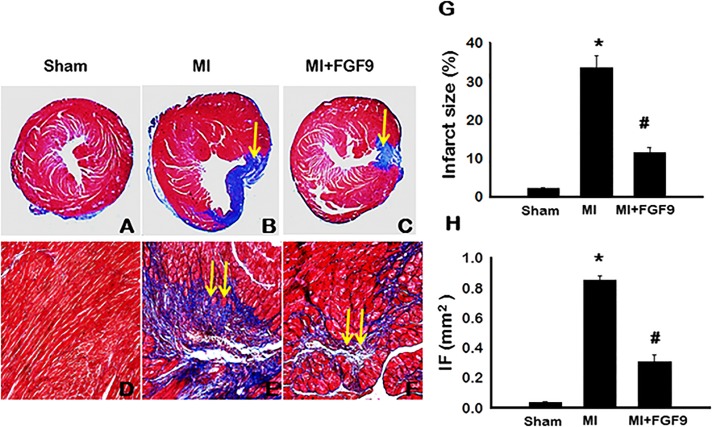
FGF-9 Inhibits Infarct and Fibrosis in the Post-MI Diabetic Myocardium. **A-F**: Representative images of Masson’s trichrome stained sections are shown depicting infarct (**A-C**) and interstitial fibrosis (**D-F**) for control and experimental groups 2 weeks post-MI. Single arrows represent infarcted myocardium. Double arrows represent interstitial fibrosis. **G**: Histogram of quantitative infarct size analysis suggests a significant decrease in infarct size in the MI+FGF-9 group compared to the MI group. **H**: Histogram of mean left ventricular cardiac myocyte cross sectional interstitial fibrotic (IF) area (mm^2^). n = 5–8 animals/group. *p<0.05 vs. sham and #p<0.05 vs. MI.

To determine the effects of FGF-9 on infarct size and interstitial fibrosis in the post-MI diabetic heart, Masson’s trichrome staining was performed on heart sections from all study groups. Representative images depicting infarct are shown in Fig. 1A-C and interstitial fibrosis in Fig. 1D-F. Quantitative analysis revealed a significant increase in infarcted myocardium in the MI group relative to the sham-operated group (p<0.05, Fig. 1G). However, when administered FGF-9 post-MI, infarct size was significantly abrogated compared to the MI group (p<0.05, Fig. 1G). Interstitial fibrosis, quantified in the left ventricular myocardium by direct measurement of the blue area using Image J software, was significantly elevated in the MI group relative to the sham group (p<0.05, Fig. 1H). Markedly, the interstitial fibrotic region was significantly reduced following FGF-9 treatment (p<0.05, Fig. 1H). Collectively, our data suggest that treatment with FGF-9 minimizes infarct size as well as blunts fibrosis formation in the post-MI diabetic myocardium.

### Monocyte Infiltration in Post-MI Diabetic Heart is Abrogated Following FGF-9 Treatment

**Fig 2:**
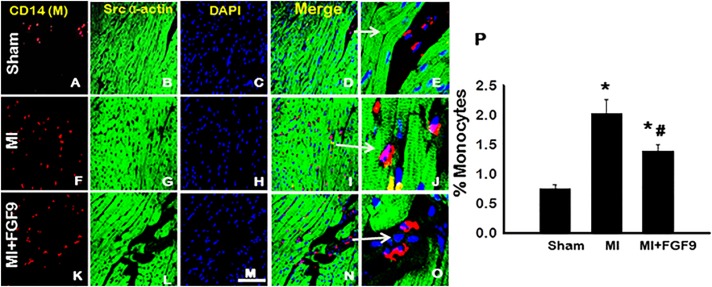
FGF-9 Blunts Monocyte Infiltration in Infarcted Diabetic Myocardium. **A-O**: Representative photomicrographs illustrate CD14 positive monocytes in red (**A**, **F**, and **K**), cardiac myocytes in green (**B**, **G**, and **L**), total nuclei in blue (**C**, **H**, and **M**), merged images (**D**, **I**, and **N**), and enhanced merged images (**E**, **J**, and **O**) for all control and experimental groups. White arrows are used to show the areas enhanced in **D**, **I**, and **N**. Scale bar = 75μm. **P**: Histogram of quantitative monocyte infiltration data 2 weeks post-MI. n = 5 animals/group. *p<0.05 vs. sham and #p<0.05 vs. MI.

To extrapolate mechanisms by which FGF-9 protects endogenous myocardial architecture post-MI, inflammatory cells, specifically monocytes, were quantified. Fig. 2A-O contains representative photomicrographs of heart sections stained with CD14, a marker for monocytes, in red (A, F, and K), sarcomeric α-actin for cardiac myocytes in green (B, G, and L), DAPI for nuclei in blue (C, H, and M), merged images (D, I, and N), and enhanced merged images (E, J, and O). Quantitative analysis suggests a significant upregulation of infiltrated monocytes following MI relative to the sham group (p<0.05, Fig. 2P). Conversely, monocyte infiltration was significantly reduced upon FGF-9 treatment compared to the MI group (p<0.05, Fig. 2P).

### M1 Macrophage Differentiation is Diminished Following FGF-9 Treatment in the Post-MI Diabetic Heart

**Fig 3:**
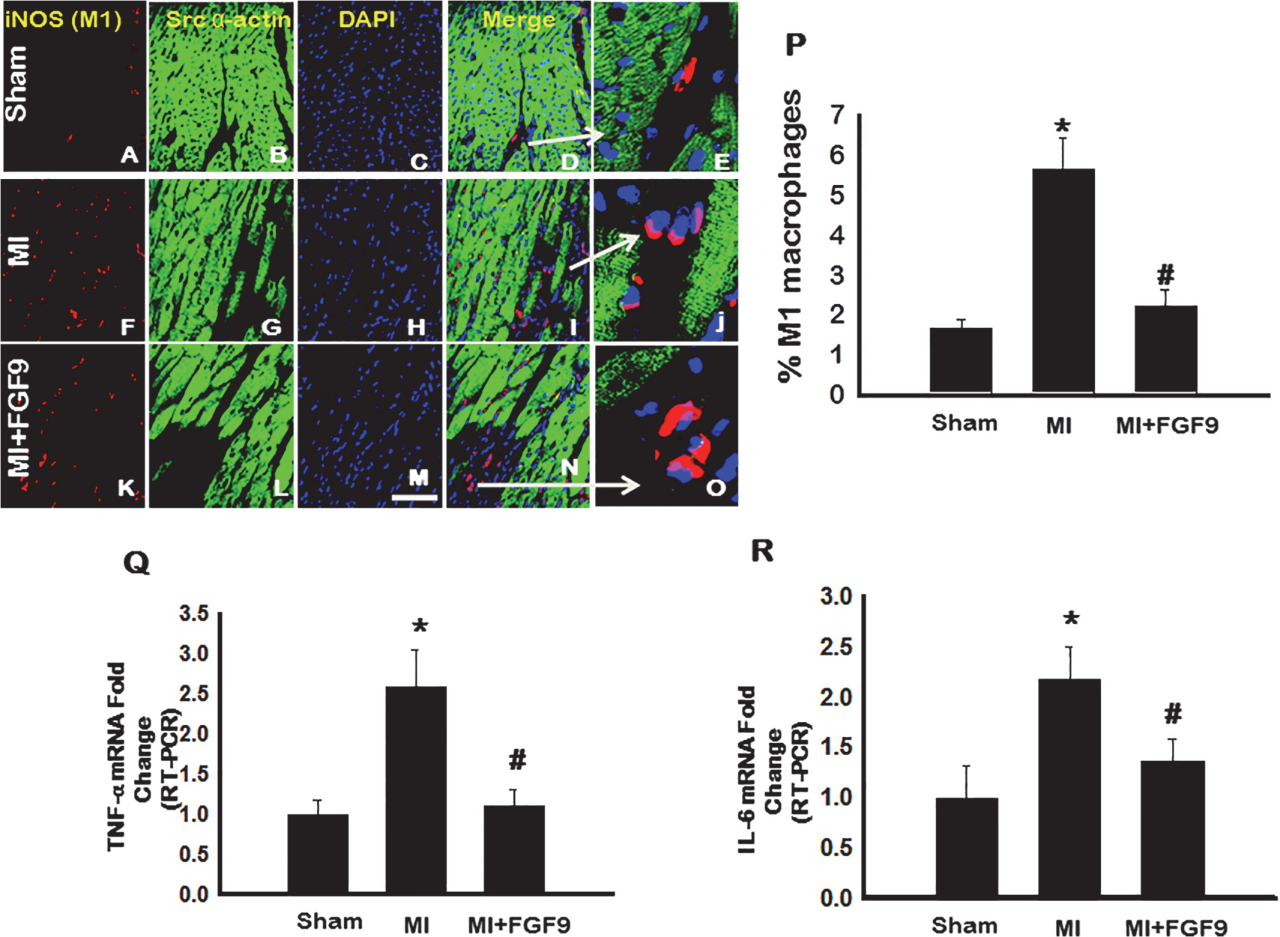
Monocyte to M1 Macrophage Polarization is Blunted Following FGF-9 Treatment. **A-O**: Representative photomicrographs demonstrate iNOS positive M1 macrophages in red (**A**, **F**, and **K**), cardiac myocytes in green (**B**, **G**, and **L**), total nuclei in blue (**C**, **H**, and **M**), merged images (**D**, **I**, and **N**), and enhanced merged images (**E**, **J**, and **O**) for all control and experimental groups. White arrows are used to show the areas enhanced in **D**, **I**, and **N**. Scale bar = 75μm. **P**: 2 weeks post-MI, M1 macrophage popoluations are significantly decreased in the MI+FGF-9 group. n = 5 animals/group. **Q**: Pro-inflammatory TNF-α mRNA expression was evaluated by RT-PCR and data provided suggest a significant increase post-MI. **R**: Proinflammatory IL-6 mRNA expression was significantly decreased following FGF-9 treatement in the post-MI diabetic heart. *p<0.05 vs. sham and #p<0.05 vs. MI.

To evaluate monocytic reprogramming outcomes post-infiltration into the infarcted myocardium, we quantified differentiated pro-inflammatory M1 and anti-inflammatory M2 macrophages. To demonstrate M1 macrophages, representative photomicrographs are shown in Fig. 3A-O depicting iNOS positive M1 macrophages in red (A, F, and K), cardiac myocytes in green (B, G, and L), total nuclei in blue (C, H, and M), merged images (D, I, and N), and enhanced merged images (E, J, and O) for all control and experimental groups. The number of iNOS positive M1 macrophages was significantly enhanced post-MI compared to sham controls (p<0.05, Fig. 3P). However, the number of M1 macrophages was significantly decreased in the FGF-9 treated mice relative to the MI group (p<0.05, Fig. 3P).

Data suggest macrophage subtypes have distinct cytokine profiles with M1 macrophages contributing to enhanced pro-inflammatory TNF-α and IL-6 secretion, to name a few [26]. In this regard, mRNA levels of TNF-α and IL-6 were evaluated by RT-PCR to identify a correlation between enhanced M1 macrophage differentiation and pro-inflammatory cytokine expression in the diabetic infarcted myocardium. Transcribed levels of TNF-α and IL-6 were significantly elevated in the post-MI heart relative to sham control animals (p<0.05, Fig. 3Q and R, respectively). Notably, post-MI administration of FGF-9 significantly blunted both TNF-α and IL-6 mRNA levels compared to the MI group (p<0.05, Fig. 3Q and R, respectively).

### M2 Macrophage Differentiation is Enhanced in the Post-MI Diabetic Heart Following FGF-9 Treatment

**Fig 4:**
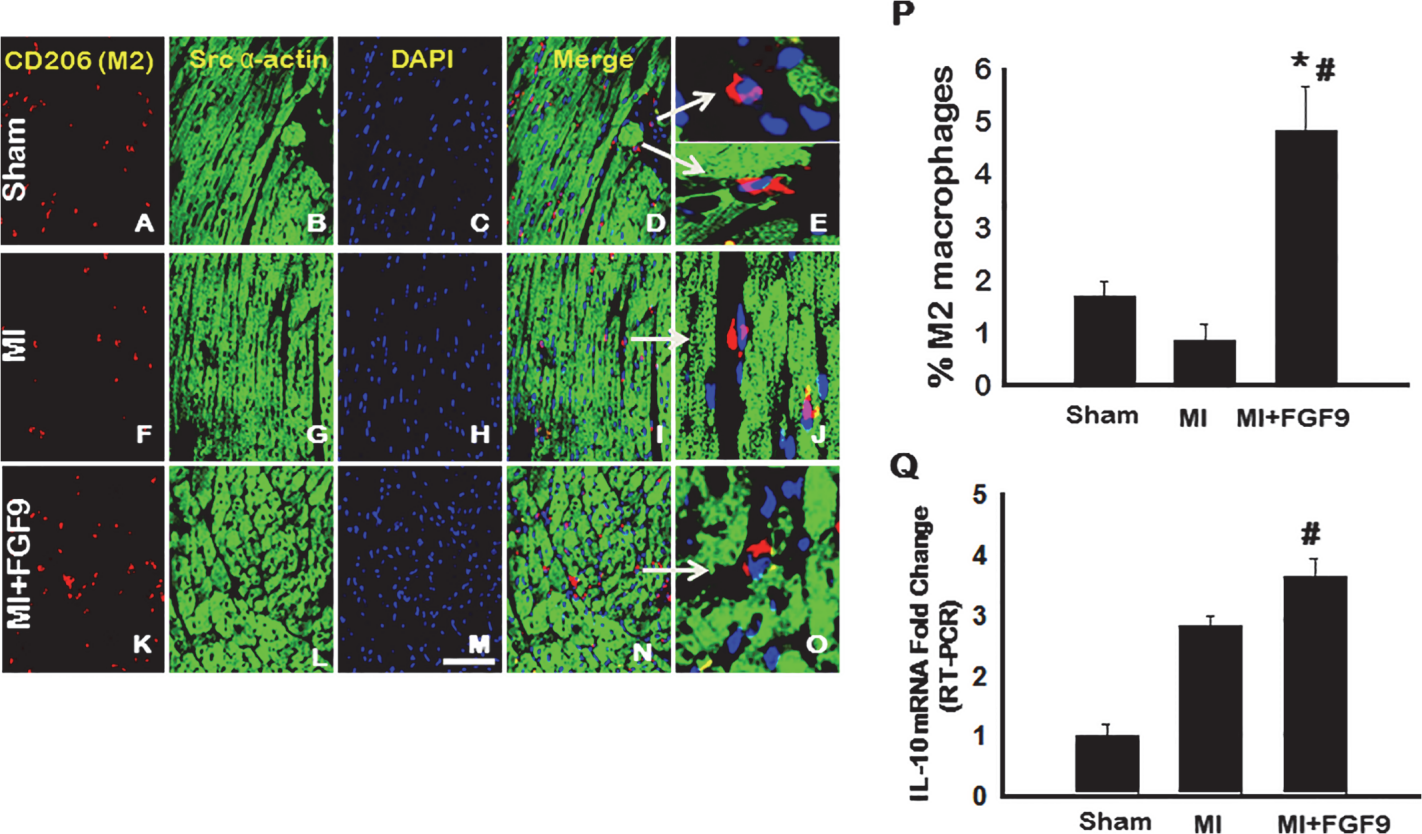
M2 Macrophages are Significantly Enhanced Following FGF-9 Treatment. **A-O**: Representative photomicrographs demonstrate CD206 positive M2 macrophages in red (**A**, **F**, and **K**), cardiac myocytes in green (**B**, **G**, and **L**), total nuclei in blue (**C**, **H**, and **M**), merged images (**D**, **I**, and **N**), and enhanced merged images (**E**, **J**, and **O**) for all control and experimental groups. White arrows are used to show the areas enhanced in **D**, **I**, and **N**. Scale bar = 75μm. **P**: Quantitative data suggest that M2 macrophage concentrations are significantly greater in the MI+FGF-9 group relative to sham and MI groups. n = 5 animals/group. **Q**: Anti-inflammatory IL-10 mRNA expression, evaluated by RT-PCR, was significantly upregulated post-FGF-9 treatment. *p<0.05 vs. sham and #p<0.05 vs. MI.

Representative images of heart sections shown in Fig. 4A-O depict CD206 positive M2 macrophages in red (A, F, and K), cardiac myocytes in green (B, G, and L), total nuclei in blue (C, H, and M), merged images (D, I, and N), and enhanced merged images (E, J, and O). Although not statistically significant, a decrease in M2 macrophage populations was observed in the MI group relative to the sham group (Fig. 4P). Importantly, results showed significantly elevated M2 macrophage concentrations in MI+FGF-9 hearts relative to sham and MI groups, suggesting FGF-9 may play a role in monocyte to M2 macrophage differentiation (p<0.05, Fig. 4P). Furthermore, mRNA expression of IL-10, an anti-inflammatory cytokine produced by alternatively activated M2 macrophages, was significantly upregulated in the MI+FGF-9 group relative to the MI group (p<0.05, Fig. 4Q) [26].

### Treatment with FGF-9 Decreases Pro-Inflammatory Cytokine Expression

**Fig 5:**
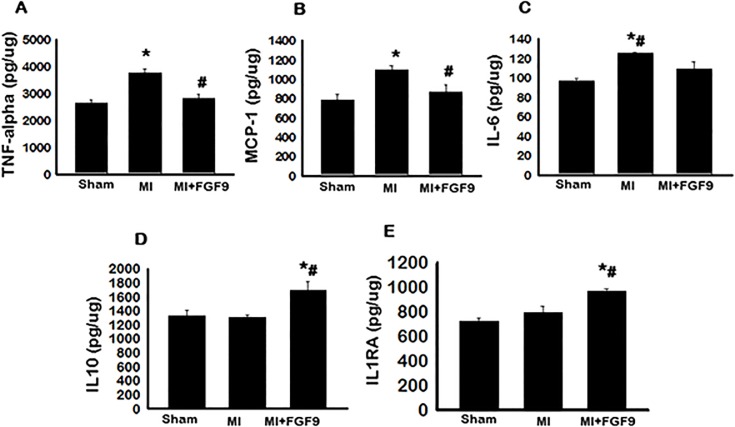
FGF-9 Treatment Augments Pro- and Anti-Inflammatory Cytokine Secretion. 2 weeks following MI, heart homogenates were used to quantify pro- and anti-inflammatory cytokine secretion via ELISA analysis. Pro-inflammatory cytokine expression, including that of TNF-α, MCP-1, and IL-6, are shown in **A**, **B**, and **C**, respectively whereas anti-inflammatory IL-10 and IL-1RA cytokine expression are depicted in **D** and **E**, respectively. n = 5 animals/group. *p<0.05 vs. sham and #p<0.05 vs. MI.

Next, we evaluated the effects of FGF-9 treatment on translated pro-inflammatory cytokine expression, including TNF-α, MCP-1, and IL-6. Quantification of TNF-α expression via a TNF-α ELISA indicated significantly escalated TNF-α levels in the MI group relative to sham controls (p<0.05, Fig. 5A). However, treatment with FGF-9 attenuated levels of TNF-α compared to the MI group (p<0.05, Fig. 5A). Results from the MCP-1 ELISA indicated increased levels of MCP-1 in the MI group as compared to the sham group (p<0.05, Fig. 5B). Conversely, the MI+FGF-9 group had significantly decreased MCP-1 expression compared to the MI group (p<0.05, Fig. 5B). Additionally, levels of IL-6 were assessed and quantitative analysis of MI tissues showed up-regulated IL-6 levels compared to sham, whereas the MI+FGF-9 group had significantly down-regulated IL-6 expression relative to expression levels in the MI group (p<0.05, Fig. 5C). Our data collectively suggest that FGF-9 promotes cardioprotection post-MI through mechanisms that contribute to down-regulated pro-inflammatory cytokine expression.

### Anti-Inflammatory Cytokine Expression Increases Following FGF-9 Treatment in the Post-MI Diabetic Heart

Reported previously, M2 macrophages secrete anti-inflammatory cytokines [27, 28]. In this regard, levels of IL-10 and interleukin-1 receptor antagonist (IL-1RA), critical mediators of inflammation and immunoregulation, were assessed. Following treatment with FGF-9, a significant increase in IL-10 was observed compared to sham and MI groups (p<0.05, Fig. 5D). Additionally, levels of IL-1RA were significantly increased in the MI+FGF-9 group compared to the sham and MI groups (p<0.05, Fig. 5E). Collectively, our data suggest FGF-9 promotes M2 macrophage differentiation and subsequent anti-inflammatory expression.

### Exogenous FGF-9 Improves Cardiac Function in Post-MI Diabetic Mice

**Fig 6:**
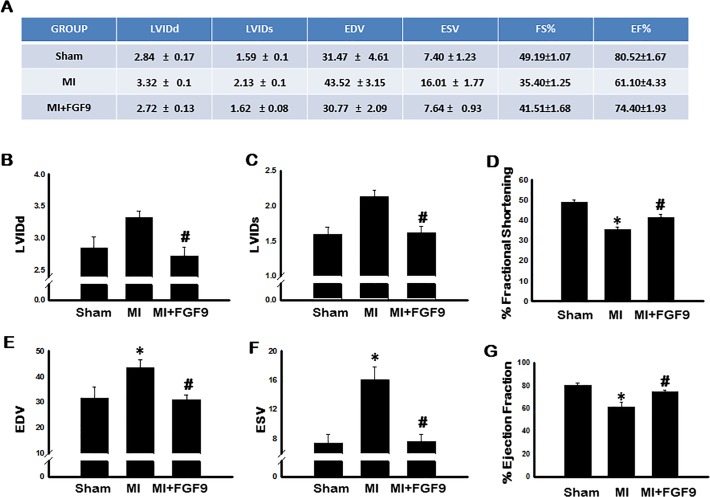
Exogenous FGF-9 Treatment Improves Cardiac Function Following MI. 2 weeks post MI, 2D transthoracic echocardiography was performed on control and experimental animals. All mean data for each quantified measurement are presented in **A**. Quantitative analyses are shown for left ventricular internal dimension-diastole (LVIDd) (**B**), left ventricular internal dimension-systole (LVIDs) (**C**), fractional shortening (FS) (**D**), left ventricular volume at end diastole (EDV) (**E**), left ventricular volume at end systole (ESV) (**F**), and ejection fraction (EF) (**G**). n = 5 animals/group. *p<0.05 vs. sham and #p<0.05 vs. MI.

To determine the impact of FGF-9 on left ventricular function post-MI, 2D transthoracic echocardiography was performed on all control and experimental mice. All raw data is provided in Fig. 6A. Two weeks post-MI, EDV and ESV were significantly increased (p<0.05, Fig. 6E and F, resepctively), whereas FS and EF were significantly diminished compared to the sham operated mice (p<0.05, Fig. 6D and G, resepctively). Notably, LVIDd, LVIDs, EDV, and ESV were significantly decreased (p<0.05, Fig. 6B, C, E, and F, resepctively) and FS amd EF were significantly improved (p<0.05, Fig. 6D and G, resepctively) in the FGF-9 treated mice relative to the MI alone mice. All echocardiographic data, taken into consideration, suggest FGF-9 preserves systolic and diastolic function and protects the diabetic heart from cardiac dysfunction consequent to MI.

## Discussion

Myocardial infarction (MI) is associated with an inflammatory response, which precedes and promotes adverse cardiac remodeling in an attempt to rescue endogenous myocardial architecture with consequential loss of normal left ventricular function [29, 30]. Monocytes are key mediators in the development and progression of inflammation as they migrate to the damaged area and differentiate into various cell types, including macrophages. Monocyte to macrophage differentiation and/or polarization yields two distinct macrophage populations: “classically activated” M1 pro-inflammatory macrophages and “alternatively activated” M2 anti-inflammatory macrophages [31]. Notably, several clinical studies have suggested the presence of elevated monocytic activity and pro-inflammatory cytokine expression, including TNF-α and IL-6, in the blood circulation of diabetic patients [32–34]. However, no studies have been carried out to examine monocyte infiltration in the diabetic post-MI heart, nor has any attempt been made to control monocyte to macrophage polarization and differentiation. Within the present study, our data, for the first time as per the best of our knowledge, will show that two weeks post-MI, there was a significant increase in infiltrated monocytes and enhanced pro-inflammatory cytokine expression in the diabetic heart. Importantly, we reported that treatment with FGF-9 1) reduced infarct size and interstitial fibrosis, 2) reduced myocardial monocyte infiltration, 3) significantly decreased M1 macrophage populations and pro-inflammatory cytokine expression, 4) enhanced M2 macrophage populations and anti-inflammatory cytokine secretion, and 5) improved cardiac function. Our data suggest FGF-9 is a novel mediator of monocyte/macrophage phenotype plasticity in the infarcted diabetic myocardium and may have therapeutic potential, with respect to a decreased inflammatory response, for the prevention of post-MI adverse remodeling.

Following MI and subsequent loss of cardiac cell types via apoptosis and necrosis, reparative processes, including fibrosis formation, are triggered to rebuild and restore infarcted tissue and maintain the architectural integrity of the myocardium [22]. Albeit necessary, fibrosis leads to an infacrt scar which lacks contractile capabilities with consequential loss of normal left ventricular funcion. Two weeks following permenant coronory artery ligation, infarcted myocardium comprised more than 30% of the total area measured with coexistent interstital fibrosis significantly elevated. Cardiac myocytes in the infacrted myocardium appeared dystrophic, enlarged, and hypertropic in nature compared to those in sham operated animals. Treatment with *ex vivo* FGF-9 significantly reduced the infarcted and interstital fibrotic area. Concurrent with our findings, Korf-Klingebiel et al. reported a significant decrease in interstitial fibrosis post-MI in cardiac-specific FGF-9 expressing transgenic mice [21].

During the initial MI-induced inflammatory response, neutrophils accumulate in the infarcted myocardium [35]. Thereafter, the myocardial filtrate is mainly comprised of monocytes and their macrophage descendants [36]. Consistent with these previous published data, monocytic infiltration was significantly increased in hearts of diabetic MI mice, as evidenced by CD14 staining. However, when additionally treated with FGF-9, monocyte infiltration was dramatically reduced. Such findings are unique in that no study has previously reported FGF-9 to play a role in the inflammatory response following MI by inhibiting the infiltration of cardiac monocytic populations.

Accruing evidence suggest that not only is monocyte infiltration a determinant of inflammation severity and disease progression but also macrophage type yields following monocyte differentiation [10, 11, 31, 36]. Specifically, studies have shown that M1 macrophages, significantly outnumbering M2 subtypes during pathological inflammation, initiate and sustain inflammation whereas M2 macrophages promote reparative processes to resolve and quench chronic inflammation [27, 35, 37]. Consistent with these investigations, M1 macrophage populations were dramatically increased with significant upregulation in secreted pro-inflammatory cytokines and M2 macrophage polarization and anti-inflammatory cytokine expression was blunted following MI surgery in diabetic mice. Conversely, following FGF-9 treatment, monocyte to M1 macrophage polarization and secreted TNF-α, MCP-1, and IL-6 were significantly reduced whereas M2 polarization and anti-inflammatory IL-10 and IL-1RA expression were dramatically enhanced. Data presented suggest FGF-9 protected the diabetic heart from post-MI remodeling by directing the differentiation of monocytes into M2 macrophages and ultimately minimizing the adverse endogenous inflammatory response.

Finally, we needed to determine the effects of FGF-9 treatment post-MI on overall cardiac function. FGF-9 treatment significantly improved left ventricular function as evidenced by enhanced EF and FS relative to the MI group. Albeit mechanisms leading to improved cardiac function are complex, our data suggest that FGF-9 may provide cardioprotection, structurally and functionally, by driving monocyte to M2 macrophage differentiation, thus minimizing adverse inflammatory-induced cardiac remodeling in the diabetic heart.

In conclusion, our data show, for the first time, that the diabetic infarcted myocardium is infiltrated with monocytes and differentiated M1 macrophages with enhanced pro-inflammatory cytokine secretion observed. Remarkably, treatment with FGF-9 significantly decreased infiltrated monocyte concentrations, M1 macrophage differentiation, and pro-inflammatory cytokine secretion. Furthermore, FGF-9 treatment increased M2 macrophage differentiation as well as the secretion of anti-inflammatory mediators ultimately protecting the infarcted diabetic myocardium from adverse cardiac remodeling and improving cardiac function. Collectively, our data suggest that FGF-9 directs monocyte differentiation and has cardioprotective potential in the post-MI diabetic heart.
